# Obstructive Sleep Apnea in a Patient with Cornelia de Lange Syndrome

**DOI:** 10.7759/cureus.1993

**Published:** 2017-12-28

**Authors:** Saif Mashaqi, Jill Hennessy, Matthew Eaton, Joel Erickson

**Affiliations:** 1 Sleep Medicine, University of north dakota; 2 Sleep Medicine, Sanford Health; 3 Sleep Medicine, Valley Dental Sleep Therapy; 4 Department of Psychiatry and Behavioral Medicine, University of north dakota

**Keywords:** cornelia de lange syndrome, sleep disturbances, obstructive sleep apnea, polysomnography, sleep related hypoventilation

## Abstract

Cornelia de Lange syndrome (CdLS) is a rare genetic disorder that is characterized by specific facial, skeletal, and behavioral features associated with variable degrees of intellectual disabilities. Sleep disturbances have been reported in patients with CdLS including insomnia, sleep-disordered breathing, intrinsic sleep disorders, and circadian rhythm disorders. The prevalence of sleep-related breathing disorders, in particular, obstructive sleep apnea (OSA), was conducted using validated questionnaires. We report the first case of CdLS that confirmed a moderate to severe degree of OSA using overnight polysomnography, which is the gold standard diagnostic test for OSA.

## Introduction

Cornelia de Lange Syndrome (CdLS) is an autosomal dominant disorder with an incidence of 1:10,000 to 1:50,000 live births [1]. The clinical presentation includes various facial dysmorphism (micro brachycephaly, synophrys, long and thick eyelashes, high arched palate, micrognathia and short neck), gastrointestinal (feeding difficulties and gastro-esophageal reflux disease (GERD)), cardiovascular, neurological, and musculoskeletal (small hands and feet) abnormalities [2-3]. Over the last few decades, more genetic background has been revealed about CdLS. Genetic mutation is usually seen in at least one of three genes on chromosomes 11,13, and 25. Mutations in five genes that are the structural components of cohesion complex have been identified (NIPBL, SMC1A, SMC3, RAD21, and HDAC8) [4]. Intellectual disability is a major feature of CdLS and it ranges from mild to severe. Sleep disturbances have been noticed, but the exact prevalence is unknown. Patients with CdLS appear to have a higher risk of developing insomnia of sleep initiation and maintenance, sleep-disordered breathing, and arousal disorders. We report a patient with CdLS with moderate to severe degrees of OSA, confirmed by overnight polysomnography.

## Case presentation

A 27-year-old white male patient with CdLS was diagnosed at the age of six months. He had behavioral symptoms accompanied by an intellectual delay. The symptoms were exhibited mainly in the form of anxiety and aggression which got worse over the last year despite being on clonidine and fluoxetine. He was referred to a sleep clinic as he was concerned about his loud snoring, witnessed apnea, and excessive daytime sleepiness which were getting progressively worse over the last few years.

He usually wakes up at 7 am and retires to bed at 8 pm. He falls asleep within ten minutes, but he struggles with recurrent awakenings at night and sometimes he can’t fall back asleep. He does not feel rested in the morning and complains occasionally of morning headaches. He volunteers in a nursing home and had been told that he falls asleep at work and in public places (e.g., theaters). He does not take naps. He denied nocturnal reflux. He does not consume caffeinated beverages and does not smoke. His parents denied sleepwalking, sleep talking, night terrors, or other parasomnias. He had been gaining weight since late 2012. His body mass index (BMI) used to be at 27 kg/m2 and increased to 32 over three years. He was evaluated at the weight loss clinic and was started on phentermine which was well tolerated. He used to have insomnia of sleep initiation and maintenance, recurrent awakening at night and displayed behavioral symptoms even before the increase in BMI. Epworth Sleepiness Scale is 17/24. Parents denied any witnessed cataplexy, hypnogogic or hypnopompic hallucinations, or sleep paralysis.

On physical examination, he did not look somnolent. BMI was 32 kg/m2 and Mallampati scale IV/IV. He had a high arched palate and micrognathia, neck circumference of 16.5 inches, and no retrognathia. The rest of the physical exam was within normal limits.

**Figure 1:**
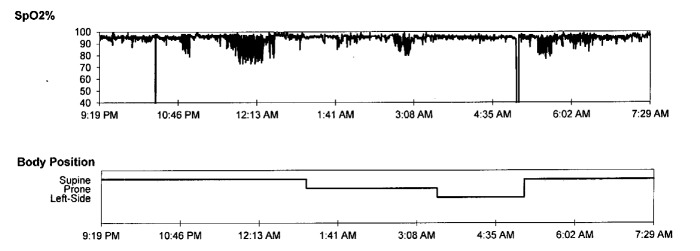
Obstructive sleep apnea with significant oxygen desaturation SpO2: peripheral capillary oxygen saturation

**Figure 2:**
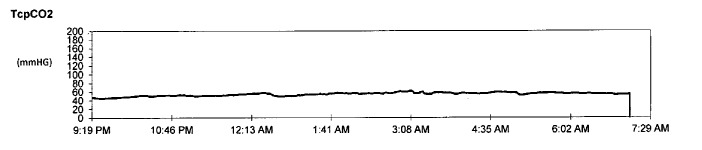
Sleep-related hypoventilation with elevated TcPCO2 readings TcPCO2: transcutaneous carbon dioxide partial pressure

He underwent overnight polysomnography which showed delayed sleep onset and elevated wake after sleep onset (WASO) time, moderate to severe OSA and sleep-related hypoventilation (Table 1). Transcutaneous carbon dioxide partial pressure (TcPCO2) was in the range of high 50’s throughout the whole night. (Figure 1-2). Spontaneous arousal index was elevated. No evidence of periodic limb movements was seen during sleep. He was given two masks to use at home for acclimation, but he did not tolerate either of them. He came back for a full night PAP titration study, which failed secondary to PAP and mask intolerance, and subsequently, he was referred to a sleep psychologist for desensitization. Unfortunately, he was not cooperative and PAP use did not improve. Accordingly, the option of mandibular advancement device (MAD) was discussed with his mother and she was in agreement to pursue this option. Unfortunately, he did not tolerate MAD secondary to anxiety. However, his mother noticed that MAD tolerance is much better than PAP therapy. A small dose of clonazepam (0.25 mg) was attempted to help with MAD tolerance.

**Table 1:** Overnight polysomnography REM: rapid eye movement AHI: apnea-hypopnea index oAHI: obstructive apnea-hypopnea index cAHI: central apnea-hypopnea index TcPCO2: transcutaneous carbon dioxide partial pressure

Sleep Architecture	
Total recording time (minutes)	610
Total sleep time (minutes)	407
Sleep efficiency (%)	67
Sleep latency (minutes)	43
REM latency (minutes)	339
Wake after sleep onset (minutes)	160
N1 (%)	15
N2 (%)	49
N3 (%)	12
REM (%)	24
Supine time (minutes)	180
Spontaneous arousal index (events/hour)	17
Cardiorespiratory Events	
AHI (events/hour)	33 (oAHI 27, cAHI 6)
REM AHI (events/hour)	7
Supine AHI (events/hour)	47
Oxygen saturation (mean, nadir) (%)	94, 71
TcPCO2 (mmHg)	(55 - 60)
Heart rhythm	Normal sinus rhythm
Limb Movements	
Periodic limb movement index (events/hour)	1.5
Periodic limb movement arousal index (events/hour)	0.3

## Discussion

Patients with CdLS appear to be at higher risk of developing sleep-disordered breathing, specifically OSA. However, all studies conducted to evaluate this risk used validated questionnaires. We report this case as the first case that showed severe OSA in a patient with CdLS using overnight polysomnography.

Some of the questionnaires used looked for non-specific sleep disturbances. Berney, et al. studied 49 patients with CdLS and found that 55% have sleep disturbances [5]. Basile, et al. used three validated questionnaires and found that 12% of 56 CdLS patients have sleep disorders with no further specification [6]. Hall, et al. found that 55% of a 54 patient sample with CdLS had sleep problems non-otherwise specified [7].

Stavinoha, et al. used three validated questionnaires that were specific for sleep-related breathing disorders in a sample of 22 patients with CdLS (The Pediatric Sleep Questionnaire (PSQ), OSA18 questionnaire, and the Pediatric Daytime Sleepiness Scale (PDSS)). Reported results show 36% of these patients had OSA [8].

Zambrelli, et al. studied 46 patients using the Sleep Disturbances Scale for Children (SDSC), which is a 26 item questionnaire that is sleep-disorder specific. They found that 17% had sleep-disordered breathing, 17% had insomnia of sleep initiation and maintenance, and 13% had arousal disorders. They also looked into the association between this scale and other features seen in CdLS (genetic mutation, GERD, BMI, intellectual functioning, epilepsy, behavioral disorders, and expressive language disorder) and found a correlation between insomnia of sleep initiation and maintenance, and GERD [9].

The overnight polysomnography conducted in our patient confirms the above findings. He had a moderate to severe degree of OSA, delayed sleep onset in the context of the clinical history which is suggestive of insomnia, sleep-wake transition disorder with elevated WASO time, and arousal disorder with elevated spontaneous arousal index. Additionally, our patient was found to have sleep-related hypoventilation based on the TcPCO2 level. To our knowledge, no studies have previously addressed this in the literature.

The exact etiology of increased risk of sleep-disordered breathing in patients with CdLS is not fully understood. It is possible that some facial features in these patients expose them to a higher risk (e.g., micrognathia, high arched palate, and short neck). Although our patient gained weight during the course of the disease and before the diagnosis of OSA, his sleep symptoms were present before the increase in BMI. A case series documented upper airway abnormalities in six patients with CdLS since these patients had reported difficulties during intubation. They found five out of six patients had laryngomalacia. Our patient did not have an upper airway evaluation [10]. We do not know the exact explanation of his sleep-related hypoventilation and whether this condition is central nervous system related or not. Our patient refused any laboratory work up, so arterial blood gas could not be obtained. Unfortunately, behavioral problems (anxiety and aggression) our patient exhibited made it difficult for him to tolerate variable treatment options. Parents denied any surgical options.

## Conclusions

This is the first case (as per our knowledge) that confirms the diagnosis of sleep-related breathing disorders in a patient with CdLS by using overnight polysomnography. We maintain a close follow-up with the patient to assess if we can re-try any of the treatment options mentioned above to see if it helps in improving his behavioral symptoms.

## References

[1] Liu J, Krantz ID (2009). Cornelia de Lange syndrome, cohesion, and beyond. Clin Genet.

[2] Jackson L, Kline AD, Barr MA (1993). de Lange syndrome: a clinical review of 310 individuals. Am J Med Genet.

[3] Kline AD, Grados M, Tuchman D (2007). Natural history of aging in Cornelia de Lange syndrome. Am J Med Genet C Semin Med.

[4] Parenti I, Gervasini C, Pozojevic J (2016). Broadening of cohesinopathies: exom sequencing identifies mutations in ANKRD11 in two patients with Cornelia de Lange-overlapping phenotype. Clin Genet.

[5] Berney TP, Ireland M, Burn J (1999). Behavioral phenotype of Cornelia de Lange syndrome. Arch Dis Child.

[6] Basile E, Villa L, Selicorni A (2007). The behavioral phenotype of Cornelia de Lange syndrome: a study of 56 individuals. J Intellect Disabil Res.

[7] Hall SS, Arron K, Sloneem J (2008). Health and sleep problems in Cornelia de Lange syndrome: a case control study. J Intellect Disabil Res.

[8] Stavinoha RC, Kline AD, Levy HP (2011). Characterization of sleep disturbance in Cornelia de Lange syndrome. Int J Pediatr Otorhinolaryngol.

[9] Zambrelli E, Fossati C, Turner K (2016). Sleep disorders in Cornelia de Lange syndrome. Am J Med Genet C Semin Med Genet.

[10] Hamilton J, Clement WA, Kubba H (2014). Otolaryngological presentations of Cornelia de Lange syndrome. Int J Pediatr Otorhinolaryngol.

